# Software implementation of systematic polar encoding based PKC-SPE cryptosystem for quantum cybersecurity

**DOI:** 10.1038/s41598-024-60767-3

**Published:** 2024-05-01

**Authors:** Ritu Redhu, Ekta Narwal, Shivani Gupta, Reena Hooda, Sonika Ahlawat, Rupali Khurana

**Affiliations:** 1 Department of Mathematics, Maharshi Dayanand University, Rohtak, 124001 India; 2 Department of Computer Science and Engineering, Indira Gandhi University, Meerpur, Rewari, Haryana India

**Keywords:** Public key cryptosystem, Polar codes, PQC, AWGN channel, Engineering, Mathematics and computing

## Abstract

The ever-growing threats in cybersecurity growing with the rapid development of quantum computing, necessitates the development of robust and quantum-resistant cryptographic systems. This paper introduces a novel cryptosystem, Public Key Cryptosystem based on Systematic Polar Encoding (PKC-SPE), based on the combination of systematic polar encoding and public-key cryptographic principles. The Systematic Polar Encoding (SPE), derived from the well-established field of polar codes, serves as the foundation for this proposed cryptographic scheme. Here, we have used MATLAB Software to introduce and implement the PKC-SPE Cryptosystem. The paper examines key generation, encryption, and decryption algorithms, providing insights into the adaptability and efficiency of systematic polar encoding in public-key cryptography. We assess the efficiency of the PKC-SPE Cryptosystem in three aspects: key size, computational complexity, and system implementation timings. In addition, we compare the PKC-SPE Cryptosystem with PKC-PC cryptosystem and find that it has reduced key sizes ($P_{r}$ = 0.8436 kbytes). The results obtained through simulations validate the effectiveness of the proposed cryptosystem and highlighting its potential for integration into real-world communication systems. Thus, in the paradigm shift to quantum computing, the PKC-SPE cryptosystem emerges as a promising candidate to secure digital communication in the quantum computing era.

## Introduction

The widely used public key cryptosystems^1^ have been broken by the rapid development of quantum computers. The discrete logarithmic and integer factorization problems serve as the foundation for the security of these public key cryptosystems^2^. But, in 1994, Peter Shor^3^ developed an algorithm that could break these public key cryptosystems. Therefore, it is necessary to replace these traditional cryptosystems^4^. In this regard, Post-Quantum Cryptography(PQC) is a promising contender that can withstand quantum computers^5^. Code-based cryptography^2^ is acknowledged as resistant to quantum computing which involves decoding a random linear code based on a hard coding theory problem in some metric. The McEliece cryptosystem^6^ has historically been the well-known cryptosystem and suffers from the drawback of having a huge public key size despite the quick encoding and decoding processes. Therefore, it is crucial to find techniques to decrease key sizes for this cryptosystem while maintaining the optimum level of security. Following the origin of this cryptosystem, researchers have proposed other variations based on error-correcting codes, including the Reed-Solomon^7^, Reed-Muller, Turbo^8,9^, and Cyclic codes. However, the majority of these variations are broken^10^ or have some flaws^11,12^ associated with them.

Polar Codes^13^, developed by Arikan in 2009, has drawn much interest as they have proven the Shannon limit of memoryless channel capacity with low coding complexity of order O(XLogX), where X is the code length. Polar Codes are based on channel polarization, and with enough recursion, the original channel tends to be either noisy or noiseless. In contrast, noiseless channels are chosen to transmit the data. Future wireless communication systems will use polar codes due to their high performance and simple complexity. It is a crucial need that 5G approaches are designed to adopt PQC for public key cryptosystems. Post-quantum cryptographic applications may benefit from polar codes because of many characteristics: It is believed that polar codes are used in various cryptosystems because of their error-correcting abilities, low complexity, and encoding and decoding algorithms that are used to reduce the size of the cryptosystem’s keys^14^. SPE algorithms combine error-correction with the advantage of maintaining the original information bits in their systematic form. By preserving these bits, error detection and correction can be improved, resulting in a lower Bit Error Rate (BER)^15^.The recursive nature of polar codes makes them suitable for real-time communication systems, cryptographic protocols, and storage technologies. As a result, polar codes are more practical for real-world applications because of their systematic nature, making a significant contribution to coding theory and cryptography^7^. In recent years, many variants^10,11,16,17^ of the McEliece encryption scheme based on polar codes have been addressed by several researchers. Kim^10^ introduced a new version of the McEliece cryptosystem^6^ using Polar Codes to increase the performance of the original McEliece encryption scheme. Later, Hooshmand et al.^11,18^ attempted to use polar codes to reduce the key length of the McEliece cryptosystem. Then, Aref^12^ proposed a non-systematic polar code-based secret key cryptosystem. Finally, PKC-PC (Public Key Cryptosystem based on Polar Codes)^19^ features an IND-CCA2 version to validate its security . These variants are based on non-systematic polar codes had reduced the key sizes upto a larger extent but still these variants are resistant against classical attacks not against quantum attacks. Our proposed cryptosystem employs Systematic Polar Encoding^20^, which is expected to be more resistant to error propagation while simultaneously reducing key size to a greater extent. Here, we have introduced Systematic Polar Encoding (SPE)^20^ in the structure of McEliece’s cryptosystem over the Additive White Gaussian Noise (AWGN) channel.

### Our contribution

The PKC-SPE cryptosystem^15^, which effectively employs Systematic Polar Encoding (SPE)^20^, is implemented to address the flaws of conventional cryptosystems. The SPE exhibits better error performance as compared to its non-systematic counterparts^20^, enhancing the robustness of the cryptosystem. The combination of simplicity, excellent error correction performance, low complexity decoding, and adaptability makes systematic polar encoding an alternative for communication systems where reliability, efficiency, and ease of implementation are important considerations. The paper aims to explore the potential of combining systematic polar encoding and public key cryptography to address the challenges of secure key exchange and confidential communication. Our paper’s main contribution is randomly selecting good bit channels to hide the generator matrix, preventing the adversary from obtaining the polar code generator matrix. The PKC-SPE cryptosystem also benefits from smaller public and private key sizes, particularly at the high-security level. Additionally, its evaluation for efficiency (implementation timings, key sizes, and computational complexity) is discussed. The key length of various blocklengths is compared with existing cryptosystems with the same security level. This paper aims to enhance security, improve efficiency, enable practical implementation, and explore potential post-quantum solutions, ultimately contributing to the advancement of cryptographic systems for secure communication.

## Implementation of the PKC-SPE cryptosystem

In this section, we will provide an overview of the processes required to construct the PKC-SPE Cryptosystem. We construct the cryptosystem by computing the Bhattacharyya parameters for a given polar code (X, K), with rate R. Bhattacharyya parameters are organized in increasing order, in which the leftmost XR indices correspond to the good bit channels (A) and the rightmost X(1-R) indices correspond to the bad bit channels. The next step is to locate the good bit channels and select the frozen bits ($A^{c}$). A submatrix $G_{AA}$ of generator matrix $G_A$ is considered to be a secret generator matrix, and the frozen bits are saved instead of the information bits. Furthermore, the secret generator matrix is also randomized using the random scrambling matrix (S), and the permutation matrix (P). By concealing the generator matrix from the opponent, the message m of length K-bits is encoded to obtain a ciphertext c of length X-bits as follows:

$$\begin{aligned} \begin{aligned} x_{A}&= u_{A}*G_{AA} + u_{A^{c}}*G_{A^{c}A}\\x_{A^{c}}&= x_{A}*inv(G_{AA})*G_{A^{c}A}\\ x&= x_{A} + x_{A^{c}} \end{aligned} \end{aligned}$$

where $u_{A} = mS$ and $u_{A^{c}}$ is taken as zero vector. This structure embeds the original information bits into the encoded sequence, which simplifies encryption and decryption algorithms. The systematic property of this algorithm makes it useful for improving error correction capabilities and securing communication channels. In the next step, simulate the encoded vector over an unsecured channel and decode the received vector. In order to determine whether or not the transmission has been successful, we validate the cryptosystem after decryption. The PKC-SPE Cryptosystem’s MATLAB code is provided below.

**Table:** 

**MATLAB implementation code of PKC-SPE cryptosystem**	
X=input(‘Blocklength’);	
display(X);	
R=input(‘Rate’);	
display(R);	
EbNo = input(‘EbNo’);	
display(EbNo);	
K = X * R;	
K = round(K);	
display(K);	
% K is blocklength	
bps = 1;	
EsNo = EbNo + 10 * $\log _{10}(bps)$;	
SNR = EsNo + 10 * $\log _{10}(R)$;	
display(SNR);	
noiseVar = 1/$(10^{(SNR/10)}$ );	
% ** Compute the Bhattacharyya parameters**	
Z = compute _ Bhattacharyya (SNR,X);	
display(Z);	
% **Find the Good Bit Channels**	
$[A,A^{c}]$ = Find _ information _ bits (Z,K);	
% **Choose the noisy channels**	
noisy _ bits = Zeros(1, X-K);	
$u_{A^{c}}$ = noisy _ bits;	
% **Generate a random message of size 1**$\times$**K**	
m = randi (2,1,K) - 1;	
$u_{A}$ = m * S;	
% S is random scrambling matrix	
% **Encode the binary input vector**	
[*u*, *x*] = polar _ code _ encoder(n,A,$u_{A}$,$A^{c}$,$u_{A^{c}}$);	
c = x * P + e;	
% P is random permutation matrix and e is intentional error vector	
% **Simulate the channel**	
y = polar _ code _ channel(X,c,SNR);	
y = y * $(P^{'})$;	
% $P^{'}$ is inverse of permutation matrix	
% **Decode the received vector**	
$u_{e}$ = polar _ code _ SC _ decoder(n,X,y,$A^{c}$);	
if (u = $u_{e}$)	
$u_{A}$ = $u_{e}(:, A)$;	
message = $u_{A} * (inv(S))$;	
%**Validation of Algorithm**	
if (m == message)	
disp(‘transmission successful’);	
else	
disp(‘transmission failure’);	
end	
else	
disp(‘transmission failure’);	
end	

## Efficiency assessment

In this section, we assess the efficiency of the PKC-SPE cryptosystem by evaluating the key size, implementation timings (key generation, encryption, and decryption), and computational complexity. These metrics not only provide insights into the performance of the cryptosystem but also play a significant role in evaluating its security against quantum attacks. Quantum computers pose a threat to traditional public key cryptosystems using algorithms like Shor’s algorithm to factor large numbers or solve the discrete logarithm problem efficiently. A smaller key size ($P_{r}$, $P_{b}$ = 0.8436, 65.25) for polar code (2048, 1741) in PKC-SPE indicates efficient use of the cryptosystem, reducing storage requirements, and secure communication over noisy channels. However, it’s essential to reduce key size with security considerations to ensure adequate protection against both classical and quantum attacks. Also, quantum computers have the potential to perform certain types of calculations exponentially faster than classical computers. Therefore, evaluating the computational complexity of PKC-SPE is crucial for assessing its security against quantum attacks. Lower computational complexity implies faster encryption and decryption algorithms, which are essential for real-time applications. Additionally, reduced complexity can lead to lower energy consumption and hardware requirements, making PKC-SPE more practical for resource-constrained environments. System implementation timings provide insights into the performance of PKC-SPE under different computational environments and also demonstrate practical efficiency in real-world applications. With efficient implementations, quantum-resistant cryptography can be seamlessly integrated into existing infrastructures and become widely adopted. Thus, it’s essential to explore the trade-offs between key size, computational complexity, and system implementation timings in the context of PKC-SPE.

### Key length

Both the public and private keys in the proposed cryptosystem are generated as follows:

- The proposed cryptosystem’s private key is given by set $P_{r}$ = $\{A^{c}, P\}$^18^, where the set $A^{c}$ is being saved in place of generator matrix $G_{A}$. These bit channel indices require $\log _2 (X)$ bits to save in binary form. Hence, the maximum bound of memory to save $A^{c}$ is calculated as $M_{A^{c}}$ $\le$ $\log _2 (X)(X-K)$. The memory required to store the permutation matrix P is calculated as $M_{P}$ = $\log _2 (X!)$. Therefore, the maximum bound of memory required to store the private key is calculated as $M_{Pr}$ = $M_{A^{c}}$ + $M_{P}$. By taking the (1024,768) Polar Code, we obtained $M_{P_{r}} = 0. 4262$ kbytes.
- The public key ($P_{b}$) consists of the structured encryption matrix of the form $[I_{K}|T]$^18^, which requires K(X-K) bits instead of KX bits. By taking the (1024, 768) Polar Code, we obtained $M_{P_{b}} = 24.576$ kbytes.

**Figure 1:**
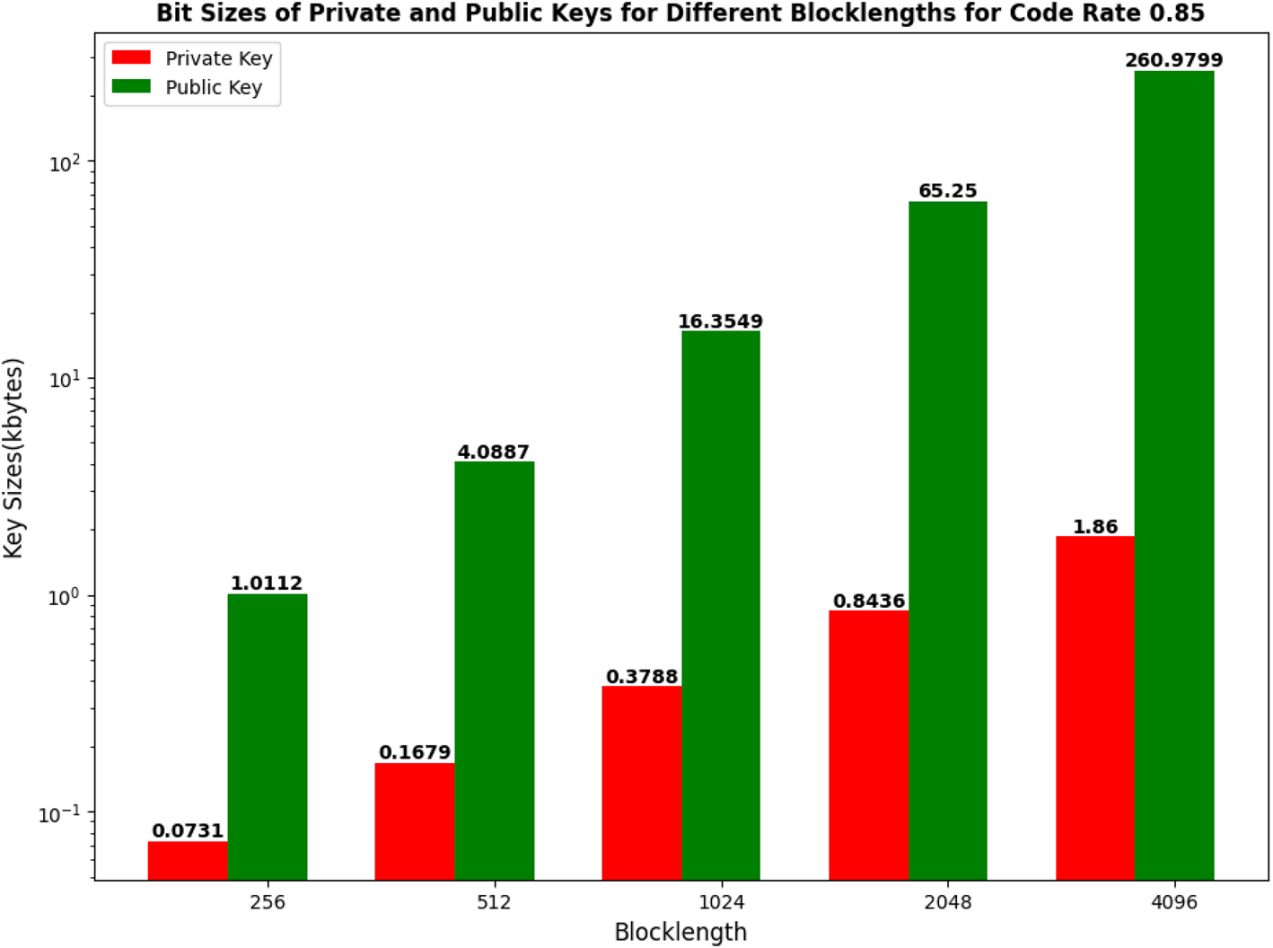
Key sizes (kbytes) vs different blocklengths for code rate 0.85.

The bit sizes of both the private and public keys for different blocklengths are being shown in Table 1 and Fig. 1.

**Table 1:** Public keys and Private keys for different blocklengths for code rate 0.85.

Blocklength	$P_{r}$(kbytes)	$P_{b}$(kbytes)
256	0.0731	1.0112
512	0.1679	4.0887
1024	0.3788	16.3549
2048	0.8436	65.2500
4096	1.8600	260.9799

### Computational complexity

The proposed cryptosystem’s complexity is divided into two parts: encoding complexity$$(C_{enc})$$ and decoding complexity$$(C_{dec})$$. The cryptosystem is being encrypted by calculating the product $$mG_{A}$$ and by adding the intended error vector e. As a result, the encoding complexity is defined as $$C_{enc} = C(mG_{A}) + C(e)$$, where $$C(mG_{A})$$ = O(K(X-K)) is the binary operations required to obtain the product$$(mG_{A})$$ and C(e) = X is the binary operations required to add X-bit intended error vector. Similarly, the decoding complexity can be expressed as$$C_{dec}$$ = C$$(yP^{-1})$$ +C$$(y^{-1})$$+C$$(u_{A^{c}})$$, where C$$(yP^{-1})$$ = O(X) is the binary operations required to calculate $$(yP^{-1})$$^11^. Furthermore, the complexity of SC decoding is calculated as C$$(y^{-1})$$ = O(X$$\log$$X) and O($$K^{2})$$ is the binary operations required to obtain $$u_{A}$$. Thus, C($$u_{A}$$)= O($$K^{2})$$. The Table 2 shows the encryption and decryption complexity of the proposed cryptosystem.

**Table 2:** Complexity of the proposed cryptosystem.

Cryptosystem	Complexity	
	Encryption complexity	Decryption complexity
PKC-SPE	O(K(X-K) + X)	O($X^2 + XLogX +K^2$)

### Implementation timings

**Figure 2:**
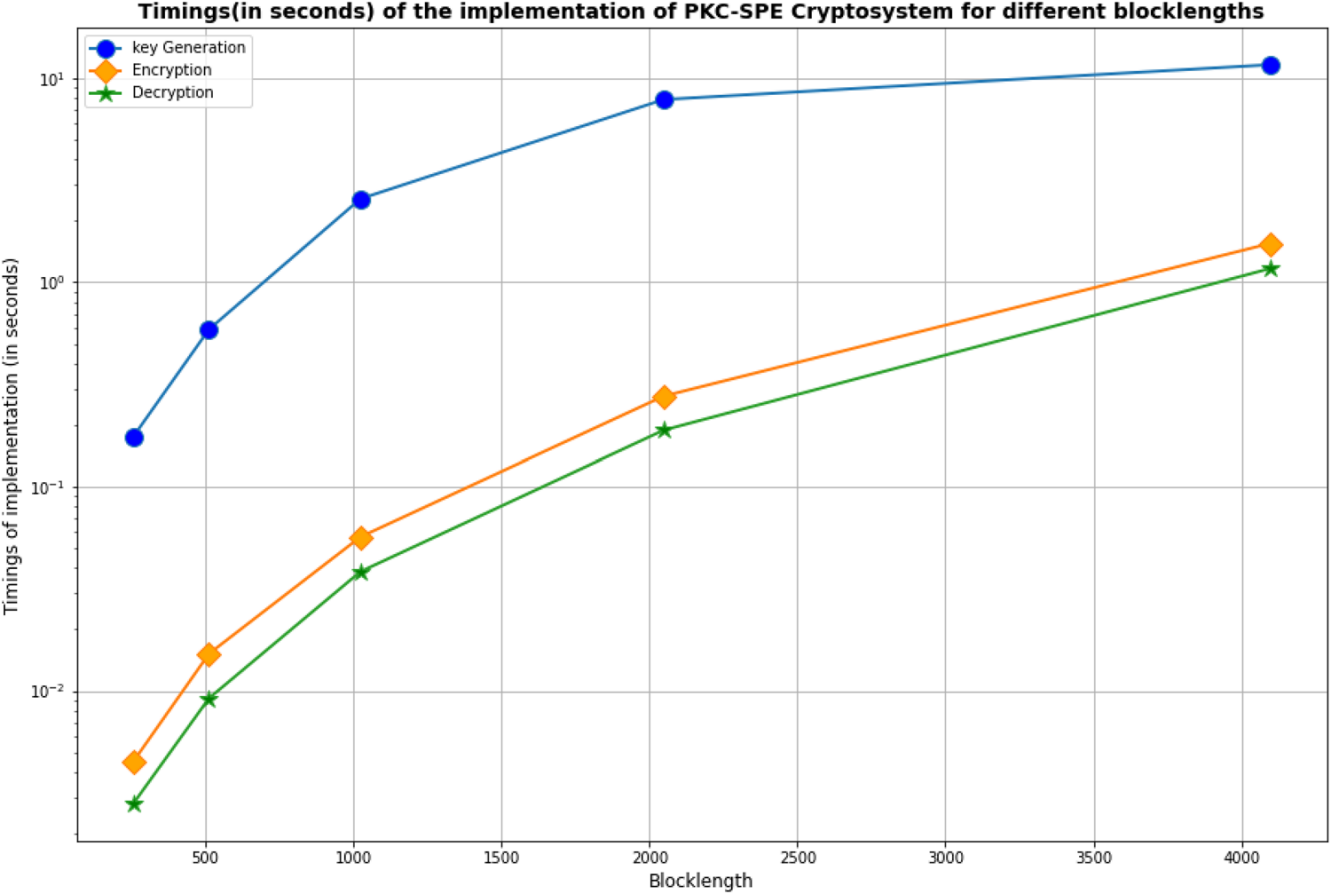
Implementation timings (in seconds) vs different blocklengths.

We have implemented the proposed cryptosystem on an 11th Gen Intel (R) Core(TM) $i5-1135G7 @2. 40GHZ$ processor. This part presents implementation timings (Key Generation, Encryption, and Decryption timings) for various blocklengths using the MATLAB Software^21^. The results for each blocklength are computed by executing 1000 random messages and averaging the execution time. Table 3 and Fig. 2 shows our cryptosystem’s implementation timings (in seconds) for various blocklengths. From Fig. 2, we find that the decryption algorithm takes least time in the PKC-SPE cryptosystem.

**Table 3:** Implementation timings (in seconds) of PKC-SPE Cryptosystem for different blocklengths.

Blocklength	Key generation	Encryption	Decryption	Total time
256	0.175	0.0045	0.0028	0.1823
512	0.5874	0.0152	0.0092	0.6118
1024	2.5615	0.0568	0.0384	2.6567
2048	7.8485	0.2765	0.1884	8.3134
4096	11.6185	1.5467	1.1650	14.3302

### Ethics approval

This article does not contain any studies with human participants or animals performed by any of the authors.

## Comparison with similar existing schemes

**Figure 3:**
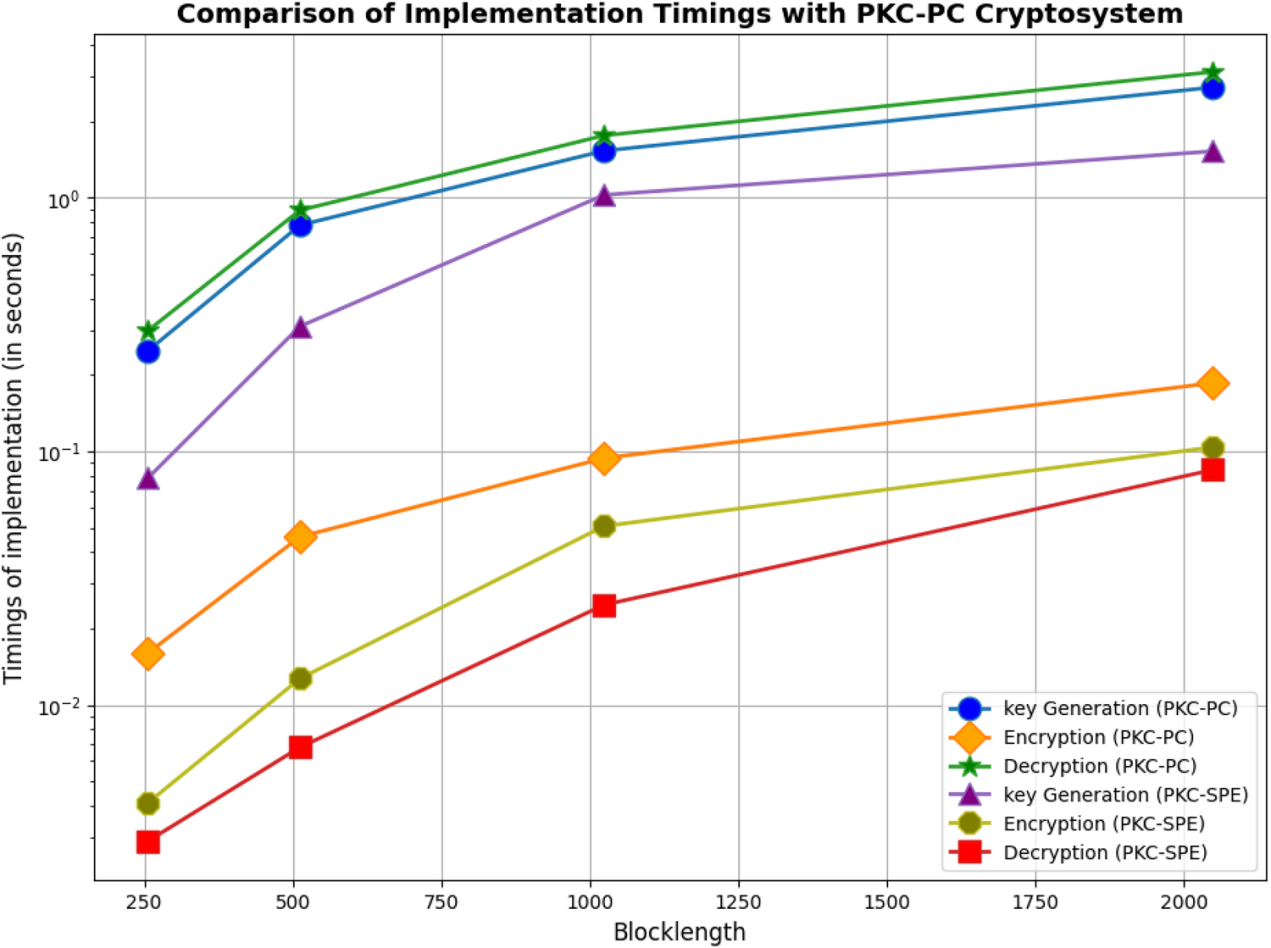
Comparison of implementation timings of different blocklegths.

The proposed PKC-SPE cryptosystem is being compared to existing schemes^14^ in this section. This section evaluates the efficacy and uniqueness of the PKC-SPE cryptosystem against similar existing schemes. The strength and practicability of our PKC-SPE cryptosystem are evaluated by evaluating several factors such as key size, computational complexity, encryption and decryption speed, key generation timings, etc. Several relevant tables and figures illustrate the comparison of PKC-SPE to similar existing cryptosystems. In Table 4, we compare key lengths with existing cryptosystems and find that our proposed cryptosystem has shorter key lengths. Table 5 presents the theoretical comparison of computational complexity with McEliece and PKC-PC cryptosystem. Table 6 and Fig. 3 compare the implementation timings of key generation, encryption and decryption algorithms for different block lengths of the proposed cryptosystem, PKC-SPE and already existing PKC-PC^19^ cryptosystem.

**Table 4:** Comparing the lengths of keys for PKC-SPE cryptosystem with the existing encryption schemes.

Encryption schemes	Code	(X, K)	R	Key length (kbytes)
McEliece^6^	Goppa	(1024, 524)	0.51	102.5
S. Kim^10^	Polar	(2048, 1536)	0.75	384
R. Hooshmand^18^	Polar	(1024, 768)	0.75	9. 34
PKC-PC^19^	Polar	(1024, 768)	0.75	24
Proposed PKC-SPE	Polar	(1024, 768)	0.75	0.4262

**Table 5:** Theoretical comparison of computational complexity with McEliece and PKC-PC cryptosystem.

Cryptosystem	Complexity	
	Encryption complexity	Decryption complexity
McEliece	O(XK)	O($X^2 + Xt +K^2$)
PKC-PC	O(K(X-K))	O($X^2 + XLogX +K^2$)
PKC-SPE	O(K(X-K) + X)	O($X^2 + XLogX +K^2$)

**Table 6:** Comparison of implementation timings with PKC-PC cryptosystem.

Cryptosystem	Timings			
	Key generation	Encryption	Decryption	Total time
X = 256				
PKC-PC	0. 249	0. 016	0. 025	0. 29
PKC-SPE	0. 0784	0. 0041	0. 0029	0. 0854
X = 512				
PKC-PC	0. 780	0. 046	0. 067	0. 893
PKC-SPE	0. 311	0. 0127	0. 0068	0. 3305
X = 1024				
PKC-PC	1. 533	0. 094	0. 135	1. 762
PKC-SPE	1. 0266	0. 0507	0. 0248	1. 1021
X = 2048				
PKC-PC	2. 728	0. 186	0. 224	3. 138
PKC-SPE	1. 5265	0. 1038	0. 0845	1.7148

Figure 3 illustrates the comparison of implementation timings between the PKC-SPE and the PKC-PC cryptosystem that demonstrates the effectiveness of PKC-SPE in terms of key generation, encryption and decryption timings. The figure depicts that PKC-SPE exhibits lesser implementation timings as compared to PKC-PC, indicating the efficiency and performance of PKC-SPE in real-world applications. Thus, the results validate the choice of systematic polar encoding in PKC-SPE and demonstrates its practical viability as a promising cryptographic solution.

## Conclusion

This paper is an improved version of the McEliece Cryptosystem that uses Systematic Polar Encoding (SPE) in the framework of a public key cryptosystem. The PKC-SPE Cryptosystem is implemented using the MATLAB Software, and its efficiency is evaluated in three aspects: key size, computational complexity, and system implementation timings. The efficiency of the software implementation has been thoroughly examined, emphasizing the cryptosystem’s security robustness, computational efficiency, and overall suitability for real-world applications. On comparing with the existing schemes, our cryptosystem has a smaller key size ($P_{r}$ = 0.8436 kbytes). Thus, systematic polar encoding has shown its ability to enhance error correction capabilities, ensuring reliable and robust communication over noisy channels. In addition, we compare it to previously known schemes, and figures and tables are presented to highlight the uniqueness and efficiency of the cryptosystem. Hence, the proposed cryptosystem, PKC-SPE provides a promising avenue for strengthening information security foundations in our increasingly interconnected digital world.

### Real life applications

In real-world applications, PKC-SPE holds immense potential across various domains such as cybersecurity, financial transactions, secure communication, and healthcare systems. Security platforms, email encryption, Virtual Private Networks (VPN), and Internet of Things devices are part of this platform. To ensure the integrity and confidentiality of financial transactions, PKC-SPE can be integrated into payment systems, digital wallets, and banking infrastructure. In addition, PKC-SPE can enhance data encryption, access control, and integrity by integrating into cloud security frameworks. Thus, PKC-SPE stands as a cornerstone of post-quantum cryptographic algorithms by enabling secure communication and data exchange in an interconnected world.

### Future scope

The cryptographic strength will be analyzed in the future by analyzing the resistance of PKC-SPE to convolutional attacks like Brute-Force attacks, Rao-Nam attacks, Information Set Decoding attacks, and so on. This will show the potential of our proposed scheme against the threats associated with quantum computing and will prove it a robust framework for secure communication.

## Data Availability

The authors declare that the data supporting the findings of this study are available within the article.

## References

[1] Narwal ER, Niram D (2023). ERN cryptosystem for the security of textual data based on modified classical encryption techniques. Indian J. Sci. Technol..

[2] Balamurugan C, Singh K, Ganesan G, Rajarajan M (2021). Code-based Post-quantum cryptography. Multidiscipl. Preprint Platform.

[3] Shor, P. W. Algorithms for Quantum Computation: Discrete Logarithms and Factoring. in *35th Annual Symposium on Foundations of Computer Science*, 124–134. 10.1109/SFCS.1994.365700. (1994)

[4] Ritu, N., Narwal, E. & Gill, S. A novel cipher technique using substitution and transposition methods. in *Rising Threats in Expert Applications and Solutions. Lecture Notes in Networks and Systems*, 123–129. 10.1007/978-981-19-1122-4-14 (2022).

[5] Khurana R, Narwal E (2023). Analysis of code-based digital signature schemes. Int. J. Electr. Comput. Eng. (IJECE).

[6] McEliece, R. J. A public-key cryptosystem based on algebraic coding theory. in *DNS Progress Report*, 114–116 (Jet Propulsion Laboratory, 1978). 10.1109/JPHOT.2021.3069510.

[7] Liu J, Wang Y, Yi Z, Lin Z (2019). polarRLCE: A new code-based cryptosystem using polar codes. Secur. Commun. Netw..

[8] Glavieux, B. A. & Thitimajshima, P. Near shannon limit error correcting coding and decoding: Turbo-codes. 1. in *IEEE International Conference on Communications, 1993. ICC ’93*. Technical Program, Conference Record, 2, 1064–1070 (1993).

[9] Sobhi Afshar AA, Eghlidos T, Aref MR (2009). Efficient secure channel coding based on quasi-cyclic low-density parity-check codes. IET Commun..

[10] Shrestha, S. R. Design of new public key encryption scheme based on the polar coding. in *Proceedings of the Twenty-Third International Joint Conference on Artificial Intelligence (JCCI’13)* (2013).

[11] Reza, H., Mohammad, S. K., Taraneh, E. & Mohammad, A. R. Reducing the key length of McEliece cryptosystem using Polar Codes. in *Proceedings of ISCISC*, 104–108 (2014).

[12] Reza H, Taraneh E, Mohammad AR (2015). Secret key cryptosystem based on non-systematic PC. Irel. Pers. Commun..

[13] Arikan E (2009). Channel polarization: A method for constructing capacity-achieving codes for symmetric binary-input memoryless channels. IEEE Trans. Inf. Theory.

[14] Redhu R, Narwal E (2023). Polar code-based cryptosystem: Comparative study and analysis of efficiency. Indones. J. Electr. Eng. Comput. Sci..

[15] Redhu R, Narwal E (2024). PKC-SPE: A variant of mceliece cryptosystem based on systematic polar encoding. Int. J. Comput. Sci. Math..

[16] Mafakheri B, Eghlidos T, Pilaram H (2017). An efficient secure channel coding scheme based on polar codes. ISC Int’l J. Inf. Secur..

[17] Khayami H, Eghlidos T, Aref MR (2022). A joint encryption-encoding scheme using QC-LDPC codes based on finite geometry. Int. J. Sci. Technol..

[18] Hooshmand, R., Shooshtari, M. K., & Aref, M. R. Secret key cryptosystem based on polar codes over binary erasure channel. in *International ISC Conference on “Information Security and Cryptology (ISCISC)*, 1–6 (2013).

[19] Hooshmand R, Shooshtari MK, Aref MR (2020). PKC-PC: A variant of the McEliece public-key cryptosystem based on polar codes. IET Commun..

[20] Wang X (2019). An optimized encoding algorithm for systematic polar codes. EURASIP J. Wirel. Commun. Netw..

[21] Hooshmand, R., Naserizadeh, F. & Mazloum, J. *Hardware Implementation of a Polar Code-based Public Key Cryptosystem*. 10.48550/arXiv.2212.13421 (2023).

